# Rare case report and literature review of peripheral T-cell lymphoma presenting as massive gastrointestinal hemorrhage: an unusual etiology demanding emergency surgical intervention

**DOI:** 10.3389/fonc.2026.1742112

**Published:** 2026-05-08

**Authors:** Zhou Liu, Liang Zhang, Qianqian Chen, Huihua He, Yuanguo Xiong, Wei Peng, Yanliang Liu, Guang Li, Liying Zhan

**Affiliations:** 1 Department of Intensive Care Unit, Renmin Hospital of Wuhan University, Wuhan, Hubei, China; 2 Department of Radiology, Renmin Hospital of Wuhan University, Wuhan, Hubei, China; 3 Department of Pathology, Renmin Hospital of Wuhan University, Wuhan, Hubei, China; 4 Department of Pharmacy, Renmin Hospital of Wuhan University, Wuhan, Hubei, China; 5 Department of Gastrointestinal Surgery, Renmin Hospital of Wuhan University, Wuhan, Hubei, China

**Keywords:** emergency endoscopy, massive gastrointestinal bleeding, partial small intestinal resection, peptic ulcer, peripheral T-cell lymphoma

## Abstract

We report a case of peripheral T-cell lymphoma (PTCL) initially presenting as idiopathic recurrent gastrointestinal hemorrhage, which had previously been misdiagnosed as multiple gastrointestinal ulcers. The recurrent hemorrhage proved to be refractory to conservative management. Unfortunately, the patient developed hemorrhagic shock due to massive gastrointestinal bleeding and was transferred to the intensive care unit (ICU). Emergency endoscopy revealed multiple ulcers in the duodenum and ileum, raising a strong suspicion for inflammatory bowel disease (IBD). However, the patient showed no significant improvement in response to corticosteroid therapy. After multidisciplinary consultation, intervention therapy was performed, but no evidence of contrast extravasation was observed. The patient continued to experience recurrent hematochezia and hematemesis, accompanied by profound anemia, with hemoglobin level dropping as low as 45 g/L. Subsequently, the patient’s level of consciousness deteriorated, and progressive hypotension developed. Given the critical condition, emergency exploratory laparotomy was performed, which finally revealed multiple longitudinal ulcers, cobblestone-appearance mucosa, and numerous active bleeding sites throughout the large lumen of the small intestine. Consequently, partial small intestinal resection with enterostomy was carried out. Histopathological examination of the resected specimen ultimately confirmed the rare diagnosis of PTCL. Postoperatively, the patient’s gastrointestinal bleeding significantly improved, and the patient was subsequently discharged. The rare case of life-threatening gastrointestinal bleeding underscores the necessity of heightened clinical awareness of PTCL to minimize misdiagnosis and delayed diagnosis, thereby facilitating timely intervention.

## Introduction

Peripheral T-cell lymphoma (PTCL) is a highly heterogeneous and relatively rare hematologic malignancy derived from mature T cells or natural killer (NK) cells, accounting for approximately 10%–15% of all non-Hodgkin lymphomas (NHLs) and roughly 20% of aggressive lymphomas (1). The most recent National Comprehensive Cancer Network (NCCN) clinical practice guideline (Version 1.2026) recommends that the diagnosis of PTCL should be based on an integrated approach combining histopathological biopsy, immunophenotyping, and molecular genetic testing owing to the frequently non-specific clinical presentation (2). The pathogenesis of PTCL is complex and has not been fully elucidated (3). It is thought to be driven by the dysregulation of key signaling pathways such as NF-κB and JAK/STAT (4), mutations in genes encoding epigenetic regulators (e.g., TP53, IDH2, TET2, and DNMT3A genes) (5), and various chromosomal abnormalities (6). According to the fifth edition of the WHO classification, PTCL is classified into more than 30 distinct subtypes based on pathological morphology, immunophenotype, and genetic/molecular characteristics (7). Common subtypes included, but are not limited to, peripheral T-cell lymphoma, not otherwise specified (PTCL-NOS; ~26%), nodal T-follicular helper cell lymphoma, angioimmunoblastic-type (nTFHL-AI; ~19%), extranodal NK/T-cell lymphoma (~10%), ALK-positive anaplastic large-cell lymphoma (ALCL; ~7%), ALK-negative ALCL (~6%), adult T-cell leukemia/lymphoma (ATLL; ~9%), enteropathy-associated T-cell lymphoma (EATL; ~5%), and hepatosplenic T-cell lymphoma (HSTL; ~1%). PTCL demonstrates considerable geographical heterogeneity in incidence and has been strongly associated with viral infections, particularly Epstein–Barr virus (EBV) (8) and human T-cell leukemia virus type 1 (HTLV-1) (9). PTCL predominantly primarily affects the elderly population, with a median age of 60 years, and demonstrates male predominance. Although PTCL can involve the gastrointestinal tract, initial presentation with related gastrointestinal symptoms is uncommon. The 5-year overall survival rate of PTCL remains poor, ranging from 30% to 50% (10).

This article reports a case of an elderly male patient who presented with recurrent high-risk gastrointestinal bleeding as the primary clinical manifestation. Endoscopy revealed multiple duodenal and ileal ulcers with active hemorrhage, raising a strong suspicion of inflammatory bowel disease (IBD). Responding poorly to conservative medical therapy, the patient subsequently developed hemorrhagic shock, impaired consciousness, and multiple organ dysfunction syndrome (MODS) due to persistent massive bleeding. Multidisciplinary consultation suggested emergency surgical exploration, which identified multiple longitudinal ulcers with active bleeding sites in the terminal ileum. Histopathology examination ultimately confirmed the rare diagnosis of PTCL-NOS. The case report highlights a rare but critical presentation of PTCL manifesting as recurrent, high-risk gastrointestinal bleeding, aiming at improving clinical awareness, facilitating earlier diagnosis and timely intervention, reducing misdiagnosis and missed diagnosis rates, and ultimately improving patient outcomes.

## Case presentation

A male patient in his late 50s was admitted to Renmin Hospital of Wuhan University in August 2025, with a 6-month history of melena and hematemesis, which had acutely exacerbated over the previous 2 days. The patient initially presented with melena of unknown etiology, followed by a sudden onset of hematemesis (approximately 200 mL), accompanied by abdominal pain, dizziness, and fatigue. He received treatment at an external hospital 1 month prior, where gastrointestinal bleeding symptoms temporarily improved. However, the symptoms recurred, characterized by tarry stools and periumbilical pain, and the patient was subsequently admitted to the hospital. The patient’s previous medical history was notable for hypertension, chronic renal insufficiency, severe anemia, and diabetes mellitus. Previous surgical history was significant for cholecystectomy performed for gallstone disease, without drug allergies, smoking, or alcohol consumption. Notably, the endoscopy performed in June 2025 revealed multiple duodenal ulcers (Stage A1), multiple gastric antral ulcers (Stage A1), and ileal ulcers.

The patient was conscious but appeared fatigued and pale. Vital signs were as follows: temperature 36.2 °C, heart rate 98 bpm, respiratory rate 32 breaths/min, and blood pressure 102/62 mmHg. No jaundice or superficial lymphadenopathy was detected. Cardiopulmonary examination was unremarkable. The abdomen was soft and non-tender without rebound tenderness. Admission laboratory findings were as follows: complete blood count, White Blood Cell (WBC) 13.21 × 10^9^/L, neutrophils 32.20%, Red Blood Cell (RBC) 2.85 × 10^12^/L, Hemoglobin (Hb) 83 g/L, Hematocrit (HTC) 21.34%, and platelets 225 × 10^9^/L; coagulation profile, Prothrombin Time (PT) 14.3 sec, Activated Partial Thromboplastin Time (APTT) 48.4 sec, fibrinogen 2.65 g/L, D-dimer 1.88 mg/L, and antithrombin III (AT-III) 56.6%; and renal and liver function and electrolytes, Alanine Aminotransferase (ALT) 8 U/L, Aspartate Aminotransferase (AST) 15 U/L, albumin 23.70 g/L, total bilirubin 10.19 μmol/L, Blood Urea Nitrogen (BUN) 6.57 mmol/L, creatinine 105 μmol/L, potassium 3.34 mmol/L, sodium 132 mmol/L, and Estimated Glomerular Filtration Rate (eGFR) 103.58 mL/min. Significantly, B-type natriuretic peptide was markedly elevated at 2,921 pg/mL with procalcitonin of 2.894 ng/mL. The blood type was A, Rh-positive. Additional tests, including cardiac injury markers, pancreatitis biochemistry, and infectious serology, were all within normal limits. Echocardiography revealed hypertensive heart disease, moderate-to-severe tricuspid regurgitation, and pulmonary hypertension. Abdominal ultrasound revealed diffuse parenchymal disease of the bilateral kidneys and mild splenomegaly. Electrocardiogram demonstrated T-wave flattening and a prolonged QT interval.

The patient’s primary diagnoses were acute gastrointestinal bleeding, peptic ulcer, and moderate anemia, complicated by hypertension, diabetes mellitus, chronic renal insufficiency, and myocardial injury. Initial treatment consisted of nothing by mouth (NPO) status with a comprehensive protocol, including acid suppression (esomeprazole 40 mg, every 12 hours), protease inhibition (somatostatin 3 mg, every 12 hours), antibiotic therapy (ceftazidime 2.0 g, every 24 hours), aggressive fluid resuscitation, parenteral nutrition support, and correction of electrolyte imbalance. On the second day, the patient suddenly developed acute abdominal pain and distension. Thus, an abdominal X-ray was performed, demonstrating step-ladder air-fluid levels consistent with partial intestinal obstruction. The fecal occult blood test was likewise positive. In response, medical therapy was intensified with aggressive acid suppression, gastrointestinal mucosal protection, and antispasmodic agents. The third day was marked by three episodes of massive melena, with a total volume of approximately 1,000 mL, accompanied by fatigue and palpitations. Continuous cardiac monitoring revealed tachycardia (114 bpm), tachypnea (18 breaths/min), and profound hypotension (64/45 mmHg). Laboratory results were significant for leukocytosis (WBC 22.31 × 10^9^/L), severe anemia (RBC 1.69 × 10^12^/L and Hb 45 g/L), thrombocytosis [platelet (PLT) 343 × 10^9^/L], and coagulopathy with low fibrinogen of 1.40 g/L. Aggressive fluid resuscitation, blood transfusion, and hemostatic agents were immediately initiated, as well as an emergent consultation. Considering the persistent acute gastrointestinal hemorrhage and hemorrhagic shock, he was urgently transferred to the intensive care unit (ICU) for further management.

**Figure 1:**
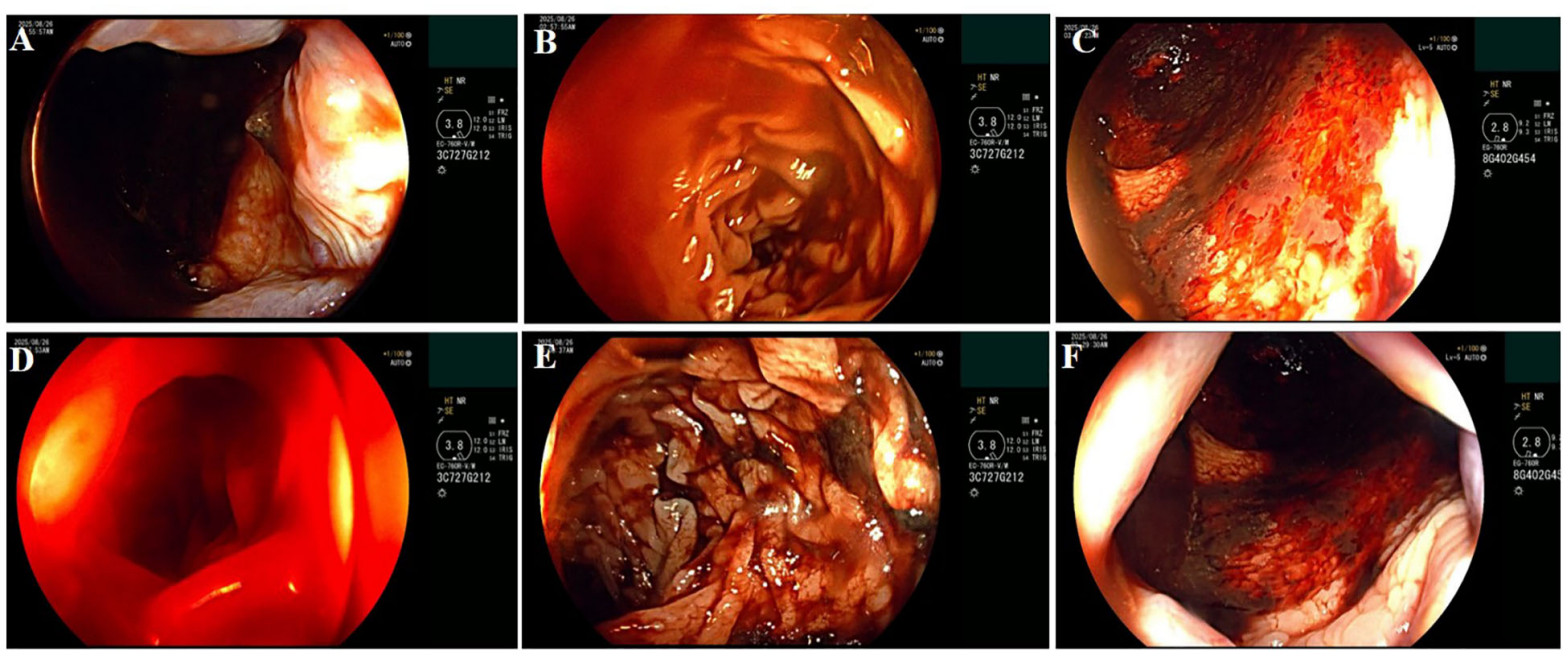
Emergency gastroscopy of the PTCL patient indicated multiple ulcers. **(A)** Bleeding in the cardia of the stomach. **(B)** Diffuse duodenal hemorrhage. **(C)** Diffuse gastritis of the gastric fundus. **(D)** Duodenal hemorrhage. **(E)** Duodenal hemorrhage and multiple ulcers. **(F)** Gastric cardia bleeding. PTCL, peripheral T-cell lymphoma.

The patient required immediate resuscitative measures, including urgent central venous catheterization, emergent blood transfusion, and initiation of vasoactive drugs. The medical regimen was modified with acid suppression (ilaprazole 40 mg, every 12 hours), protease inhibition (somatostatin 3 mg, every 24 hours), and splanchnic vasoconstriction (terlipressin 2 mg, every 24 hours). Additionally, the coagulopathy was corrected with a combination of fresh frozen plasma, human fibrinogen, and tranexamic acid. Following initial hemodynamic stabilization, the patient underwent endotracheal intubation to facilitate urgent endoscopy. As expected, endoscopy revealed a bleeding ulcer (Stage H2) on the posterior wall of the duodenal bulb (**Figure 1**). Over the subsequent day, the patient was clinically stable without further hematemesis or melena, regained consciousness, and was successfully extubated. On the fifth day, the patient experienced recurrence of melena, accompanied by a corresponding drop in hemoglobin to 51 g/L. Unfortunately, the patient experienced persistent massive bleeding after blood transfusion, with an estimated volume of 1,550 mL, causing the hemoglobin level to further decline to as low as 49 g/L.

**Figure 2:**
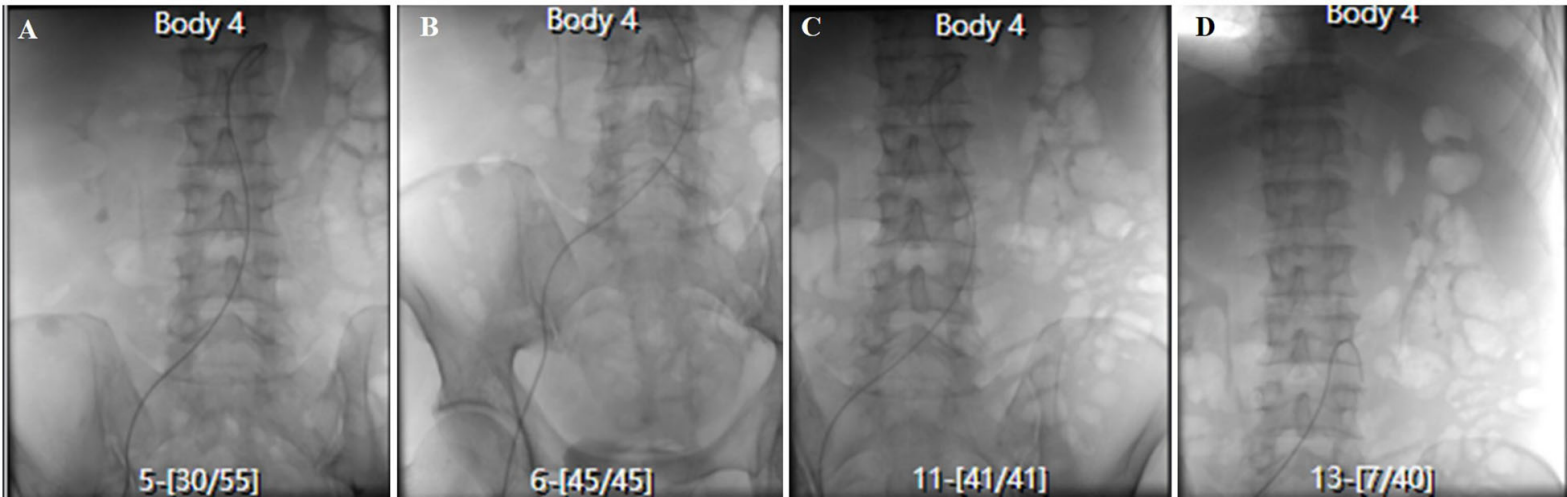
Emergency celiac and mesenteric angiogram of the PTCL patient. **(A)** Celiac artery angiography shows no abnormalities. **(B)** Gastroduodenal artery angiography shows no abnormalities. **(C)** Superior mesenteric artery angiography shows no abnormalities. **(D)** Inferior mesenteric artery angiography shows no abnormalities. PTCL, peripheral T-cell lymphoma.

**Figure 3:**
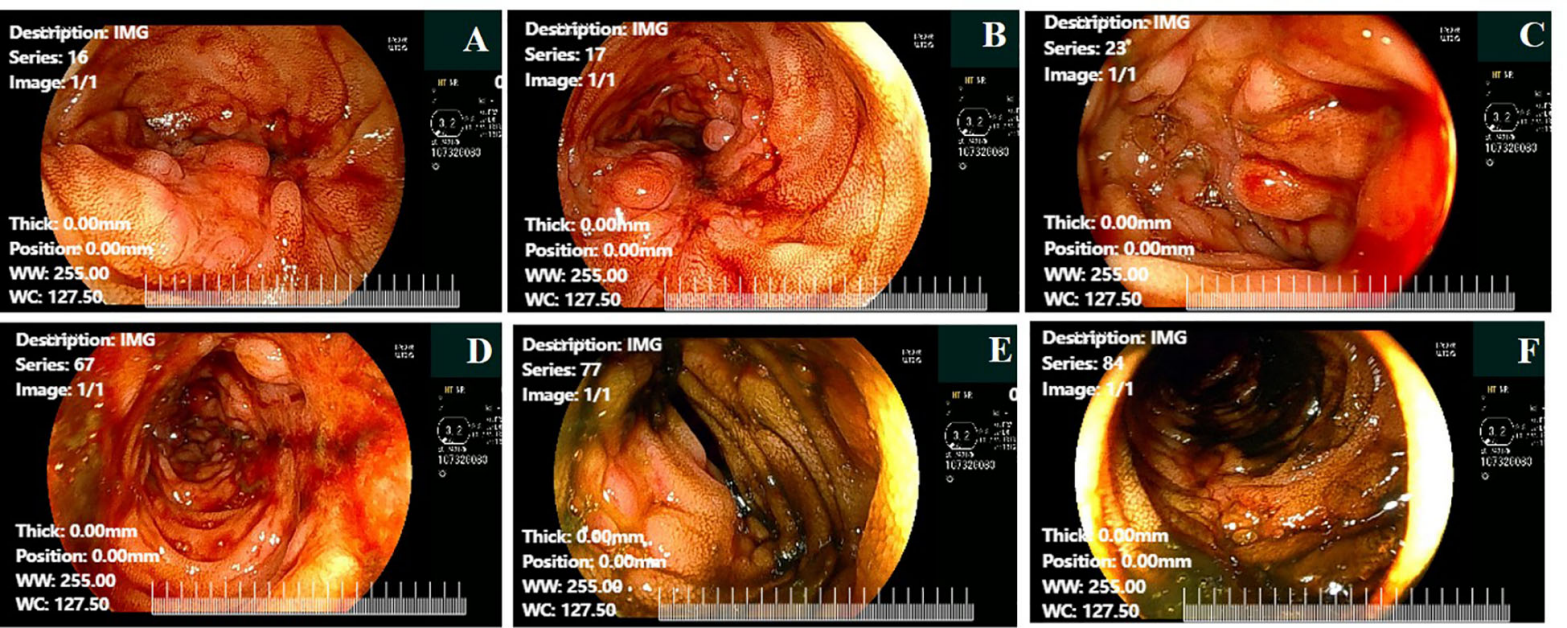
Emergency colonoscopy of PTCL patients revealed multiple ulcers. **(A)** The terminal ileum cobblestone appearance with bleeding. **(B)** The terminal ileum cobblestone appearance with bleeding. **(C)** The terminal ileum cobblestone appearance with bleeding. **(D)** The cobblestone appearance of the ileocecal valve. **(E)** The cobblestone appearance of ileocecal valve. **(F)** The cobblestone appearance of the ileocecal valve, combined with bleeding. PTCL, peripheral T-cell lymphoma.

**Figure 4:**
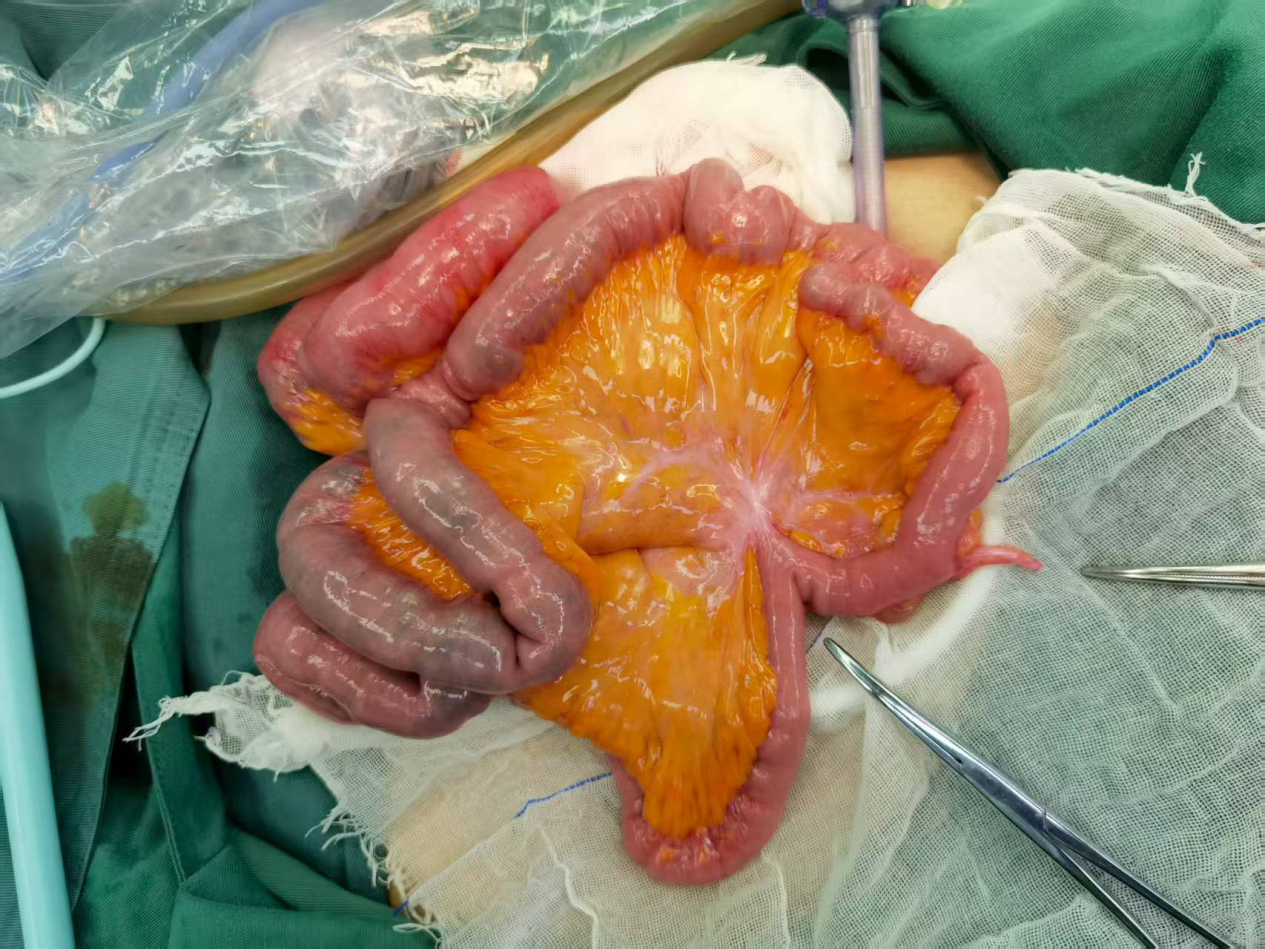
The ischemic necrosis of the small intestinal mucosa in the PTCL patient. Multiple ulcerations with swelling and scattered bleeding foci were identified in the small intestine during the operation. PTCL, peripheral T-cell lymphoma.

Following multidisciplinary consultation, emergency mesenteric and celiac angiographies were recommended and performed. Surprisingly, there was no significant contrast extravasation with just a slight contrast concentration noted in the terminal ileum (**Figure 2**). After the interventional procedure, the patient continued to have melena (approximately 600 mL) on the seventh day, with hemoglobin level continuously declining to 51 g/L. On the eighth hospital day, the patient suffered from recurrent hematochezia, prompting an urgent colonoscopy. The colonoscopy revealed multiple bleeding ulcers in the ileum, and IBD was highly considered as the possible etiology (**Figure 3**). On the ninth hospital day, empirical glucocorticoid therapy (methylprednisolone 20 mg) was initiated due to the suspicion of IBD. Subsequently, the patient developed hematochezia (approximately 500 mL), accompanied by hypotension (80/60 mmHg) and a sharp decline in hemoglobin to 43 g/L. Weighing the risks and benefits, emergency exploratory laparoscopy was immediately performed by the gastrointestinal surgeon team. To our surprise, multiple longitudinal ulcers with scattered bleeding were revealed in the small intestinal lumen, approximately 140 cm proximal to the ileocecal valve. Consequently, after careful consideration, a partial ileal resection with ileocecectomy and ileostomy was performed (**Figure 4**). Postoperatively, the recurrent gastrointestinal bleeding ceased markedly, with a gradual increase in hemoglobin levels and progressive recovery of gastrointestinal function.

**Figure 5:**
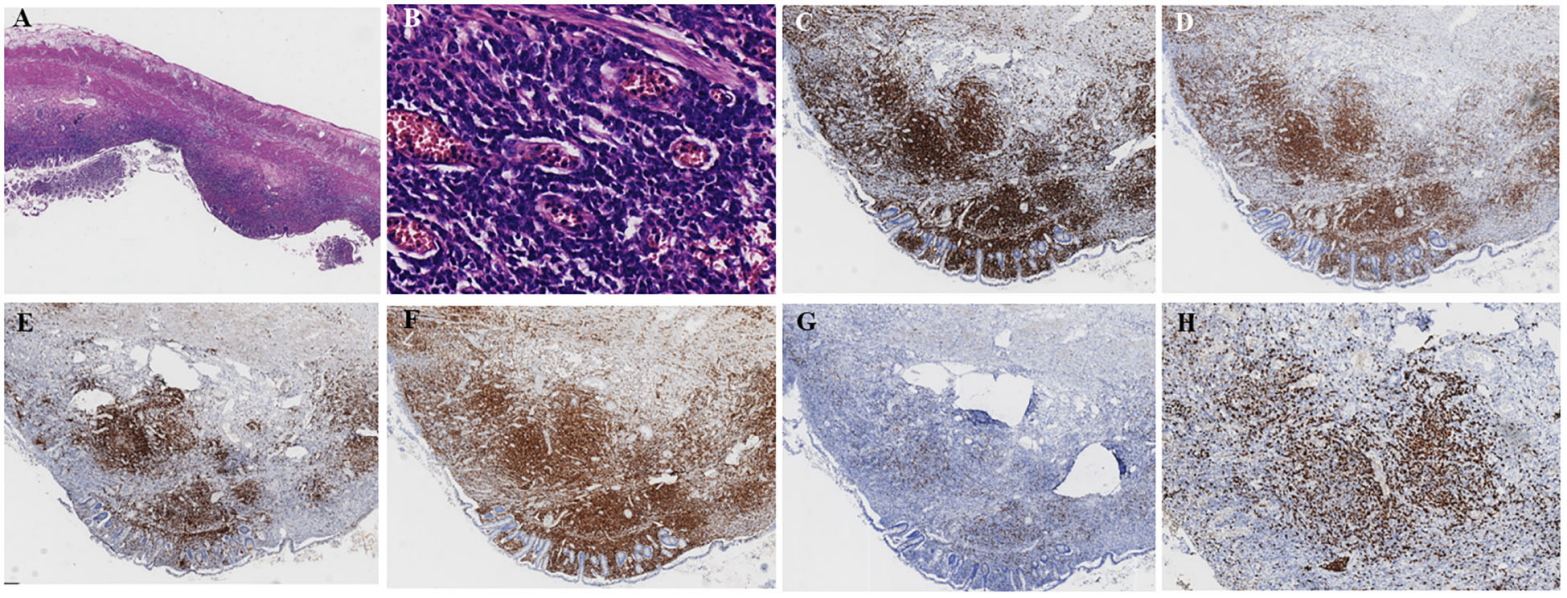
The PTCL patient’s pathological examination of the resected small intestinal biopsy. **(A)** Small intestinal ulcers with focal mucosal loss with mucosal lymphocytic infiltration (×20). **(B)** Mucosal perivascular infiltrate of monotonous atypical lymphocytes with enlarged nuclei (×200). **(C)** CD3-positive neoplastic T cells with nodular perivascular pattern (×40). **(D)** CD5-positive neoplastic T cells with nodular perivascular pattern (×40). **(E)** CD30 positivity with strong expression in ~90% of neoplastic cells (×40). **(F)** CD4 positivity in the neoplastic T cells (×40). **(G)** CD8 negativity with confirmed T-lymphocytic origin in the neoplastic cells (×40). **(H)** Ki-67 positivity with high proliferative index (~80%) in tumor cells (×100). PTCL, peripheral T-cell lymphoma.

**Figure 6:**
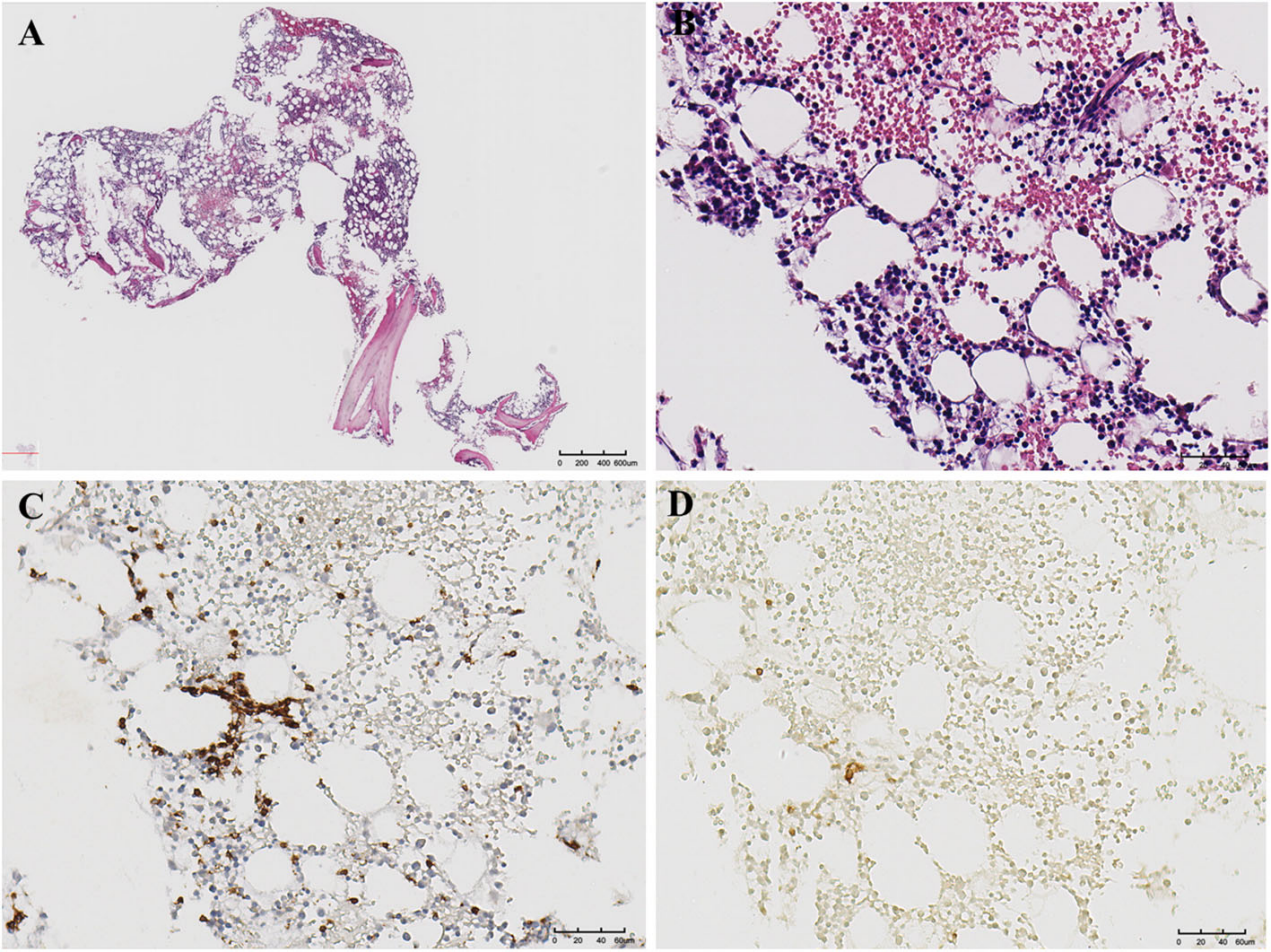
The PTCL patient’s bone marrow aspiration biopsy. **(A)** Low-magnification histopathological image of H&E (×20). **(B)** High-magnification histopathological image of H&E (×200). **(C)** Immunohistochemistry focal CD3 positive (×200). **(D)** Immunohistochemistry partial CD30 positivity (×200). PTCL, peripheral T-cell lymphoma.

**Figure 7:**
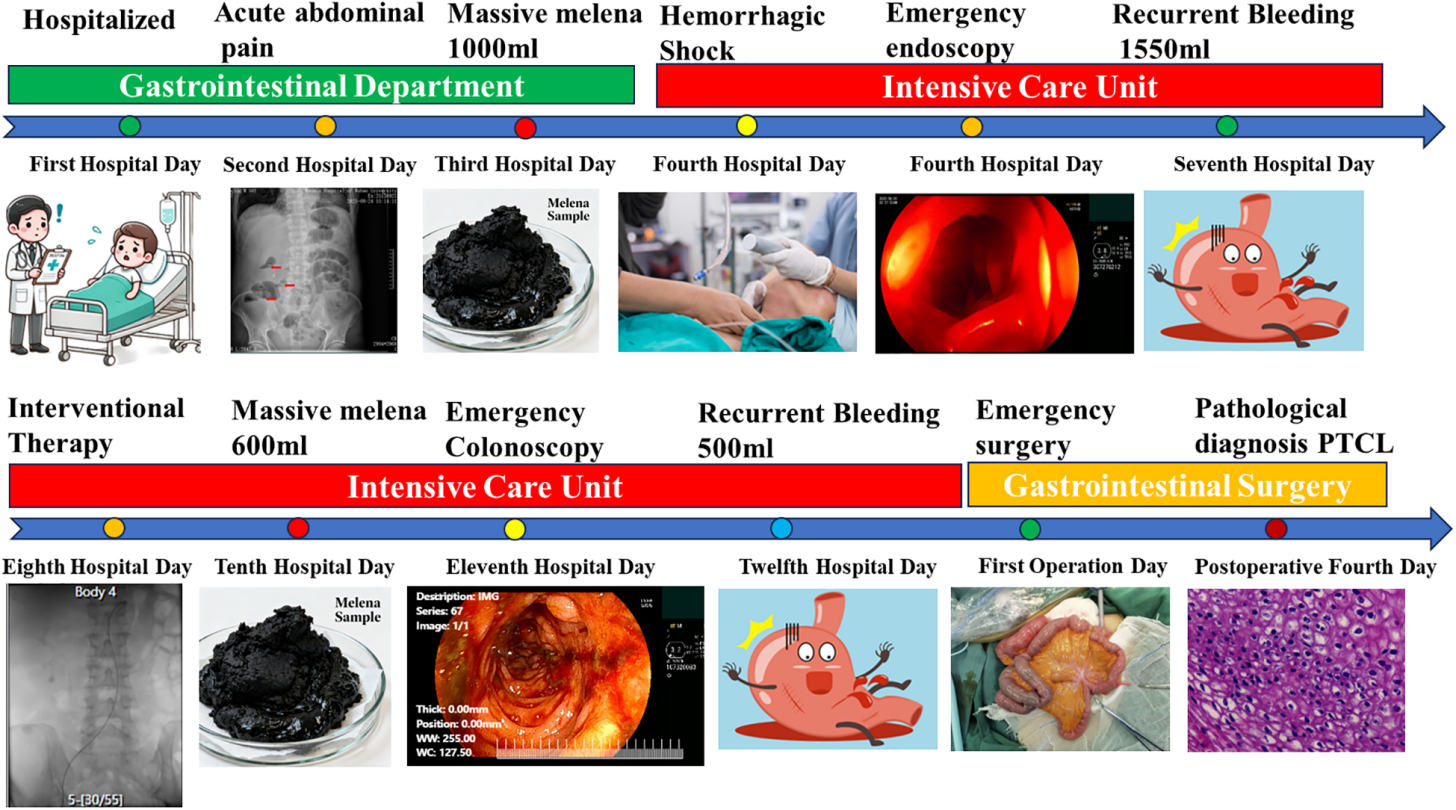
The summary flowchart depicts the evolution of the patient’s condition and essential therapeutic interventions.

Pathological examination unexpectedly revealed perivascular infiltrate by monotonous atypical T lymphocytes, leading to a definitive diagnosis of PTCL-NOS. The examination also demonstrated chronic inflammation with multifocal ileocecal ulcers and dense lymphoid infiltration. The diagnosis was supported by an immunohistochemical profile that was positive for CD3, CD5, CD30, Bcl-2, and CD4 and a high Ki-67 proliferation index of 80%, with negative stains for CD8, CD56, ALK, and EBER (**Figure 5**). On postoperative day 7, the patient strongly requested discharge against medical advice for further oncological examinations. The patient still doubted the diagnosis of malignant lymphoma, and the pathological slides were referred for external consultation 2 weeks later. The consultant described tumor cells diffusely growing around blood vessels with diffuse CD30 positivity and reconfirmed the diagnosis. The bone marrow aspiration report showed hypercellular (50%) with CD30-positive T cells, which further reconfirmed the diagnosis of PTCL (**Figure 6**). Furthermore, the PET–CT revealed postoperative changes consistent with PTCL in the terminal ileum (**Supplementary Figure 1**). A 3-month telephone follow-up confirmed that the patient was alive, with targeted CHOP therapy under consideration. The chronology of the patient’s hospital course, depicting pivotal time points, treatment modifications, pertinent diagnostic results, and intervention measures, is summarized in **Figure 7**. This case received a waiver of informed consent and was supported by the ethics committee of Renmin Hospital of Wuhan University.

## Discussions

The case report describes a middle-aged male patient hospitalized for recurrent gastrointestinal hemorrhage with multiple deep, longitudinal ulcers in the duodenum and ileum. The atypical endoscopic presentations posed considerable diagnostic dilemmas and substantially complicated clinical management. PTCL represents a rare and aggressive subtype of non-Hodgkin lymphoma with a low global incidence, although prevalence in Asia is slightly higher than in Europe (3). The clinical presentation of PTCL is non-specific and may include systemic symptoms such as unexplained fever, fatigue, weight loss, painless progressive lymphadenopathy, and frequent extranodal involvement (11). The diagnostic challenge arises not only from the non-specific endoscopic appearances, which may manifest as ulcerative, mass-forming, infiltrative, or mixed patterns, but also from anatomical and pathophysiological constraints (12–14). PTCL is a highly aggressive, typically extranodal variant characterized by insidious onset and non-specific endoscopic features. It frequently invades the full thickness of the gastrointestinal mucosa, leading to life-threatening complications such as massive hemorrhage, gastrointestinal perforation, or obstruction (15). PTCL accounts for approximately 5%–10% of lymphoma, is characterized by a high misdiagnosis rate, and is often mistaken for common diseases such as IBD (13), peptic ulcer (14), or intestinal tuberculosis (12).

The clinical manifestations are closely correlated with anatomical sites of involvement, with the small intestine being the most frequently affected. Although abdominal pain represents the predominant symptom, it is frequently accompanied by acute or chronic gastrointestinal bleeding, diarrhea, malnutrition, gastrointestinal obstruction, and perforation. Nevertheless, massive recurrent hemorrhage is seldom reported. Although rare, it has been documented by existing literature (14–17). The pathogenesis of life-threatening hemorrhage involves multifactorial mechanisms, principally categorized as direct disruption and indirect biological aggression, such as the TLR4/NF-κB and JAK/STAT signaling pathways, which promote tumor cell proliferation and inhibit apoptosis (18). The primary mechanism involves distinct tropism for diffuse transmural infiltration throughout the intestinal wall, which directly compromises the mucosal microvasculature, culminating in progressive necrosis and segmental bowel ischemia (19). In addition, biological injury contributed to the hemorrhage. 1) Cytokine storm: Activated T lymphocytes release large amounts of pro-inflammatory cytokines, such as TNF-α, γ-IFN, and interleukins (ILs), inducing apoptosis and necrosis of intestinal mucosal epithelial and vascular endothelial cells (20). 2) Protease destruction: The inflammatory response further activates the immune cells, leading to the release of matrix metalloproteinases (MMPs) and serine proteases, degrading extracellular matrix components and disrupting vascular structural integrity (21). 3) Vascular erosion: Pathologically, PTCL exhibits direct vascular erosion and infiltrates the arterial and venous walls, disrupting vascular structural integrity, increasing mucosal fragility, and elevating hemorrhage risk (22). 4) Microenvironmental effects: The tumor microenvironment promotes a hypercoagulable state, leading to micro-thrombosis and ischemic necrosis, which perpetuates a vicious cycle of hemorrhage → ischemia → necrosis → re-hemorrhage. 5) Treatment-related injury: Radiotherapy and chemotherapy can cause tumor cell destruction, with subsequent apoptosis and necrosis, exposing deep-seated ulcers and nascent capillaries, thereby predisposing to hemorrhage. Additionally, chemotherapy-induced myelosuppression results in trilineage cytopenia, which, combined with thrombocytopenia, exacerbates the pre-existing coagulopathy and bleeding risk. The patient also presented with incomplete intestinal obstruction accompanied by abdominal pain. The underlying pathophysiology may be as follows. 1) Diffuse infiltration: The infiltration of tumor cells caused marked bowel wall thickening, rigidity, and reduction of elasticity. 2) Extrinsic compression: The enlarged mesenteric or abdominal lymph nodes may have caused extrinsic compression. 3) Inflammatory response: Tumor-related multiple ulcers led to a secondary infection, which caused severe edema in the affected intestinal and surrounding tissues. These mechanisms may further exacerbate bowel wall thickening and luminal narrowing as described (23).

Although neoplasms are not the primary etiology of gastrointestinal bleeding, hemorrhagic manifestations warrant rigorous clinical attention due to the potentially poor outcomes and insidious course. The presence of “alarm” symptoms such as unexplained anemia, obstructive features, persistent localized abdominal pain, or positive fecal occult blood tests should raise suspicion for malignancy, particularly in elderly patients with chronic comorbidities. In addition to common carcinomas and other tumors (stromal tumors and neuroendocrine neoplasms), clinicians should also maintain suspicion for rare lymphomas (24). To better characterize the clinical features, subtypes, management, and outcomes of PTCL with primary gastrointestinal involvement, a systematic literature review was performed. To sum up, a total of 97 PTCL patients from seven countries are systematically summarized in **Table 1**. Consistent with epidemiology, a male predominance trend was observed (63.92% *vs*. 36.08%) with an aging population. Gastrointestinal symptoms were non-specific, mainly presented as bleeding, abdominal pain, diarrhea, and weight loss, with bleeding remaining the most predominant (14–17, 25). Multiple gastrointestinal ulcers were identified endoscopically, predominantly located in the jejunum and ileum (14, 15, 17, 26). As emphasized, obtaining adequate specimens through multi-site sampling is essential for accurate diagnosis, a point that cannot be overemphasized. Personalized treatment regimens are guided by immunohistochemical profiles and encompass chemotherapy, targeted therapy, radiotherapy, and even stem cell transplantation. Owing to the high-grade malignancy of PTCL and frequent delay in diagnosis, the prognosis has remained unsatisfactory with an overall mortality of 55.67% during follow-up.

**Table 1:** The summary information of 97 case reports on gastrointestinal peripheral T-cell lymphoma.

Research	Country	Patients	Age	Sex (M/F)	Symptoms	Endoscopic findings	Pathological findings	Diagnosis	Location	Combination	Therapy	Survival
Lu Y (14)	China	42	48 ± 26	31/11	Abdominal pain, diarrhea, gastrointestinal bleeding, weight loss, fever	Superficial, ulcerative, ulceroinfiltrative, infiltrative	Not mentioned	ENKTCL, MEITL, ALCL, PTCL	Ileocecal portion, colon, small intestine, duodenum, stomach, rectum	Perforation, hemorrhage, intestinal fistula, intestinal obstruction	Surgery, surgery + chemotherapy, chemotherapy	38.10%
Kohri M (15)	Japan	11	56–79	10/1	Tarry stool, epigastralgia, subileus, fever, diarrhea, fatigue, appetite loss	Not mentioned	CD4^+^, CD8^+^, Ki-67^Low^	EATL, ENKL, PTCL-NOS, ATL, ITLPD-GIT	Small intestine, colon, stomach	Not mentioned	Chemotherapy, radiotherapy, chemotherapy + radiotherapy	54.55%
Mitarnun W (16)	Thailand	7	Not mentioned	4/3	Fever, weight loss, anemia, gastrointestinal bleeding	Not mentioned	CD3^+^, CD15^−^, CD16^−^, CD30^−^, CD57^−^, CD68^−^	PTCL	Gastrointestinal tract	Not mentioned	Chemotherapy	28.57%
Nakaji K (21)	Japan	1	71	1/0	Gastrointestinal bleeding	Ulcer jejunum	CD3^+^, CD5^+^, CD10^−^, CD20^−^	PTCL-NOS	Jejunum	Intestinal perforation	Chemotherapy	100.00%
Tao J (24)	China	1	68	1/0	Dyspepsia, weight loss	Multiple ulcerative	CD2^+^, CD3^+^, CD5^+^, CD79a^+^, CD59^−^	PTCL-NOS	Duodenum proximal ileocecum	Massive gastrointestinal hemorrhage	Chemotherapy	0.00%
Fujii Y (25)	Japan	1	78	1/0	Gastrointestinal bleeding	Not mentioned	Not mentioned	ATLL	Jejunum	Not mentioned	Surgery	100.00%
Hiraga H (26)	Japan	1	45	1/0	Diarrhea and weight loss	Ileum ulcer	CD3^+^, CD56^−^	EATL	Ileum	Diarrhea	Chemotherapy	0.00%
Chung J (34)	Korea	1	25	0/1	Abdominal pain, hematemesis	Gastric fundus ulcer	CD30^+^, CD3^+^, TIA-1^+^, CD20^−^, CD56^−^	ENKL	Stomach	Not mentioned	Chemotherapy	100.00%
Ponnusamy R (35)	India	1	56	1/0	Abdominal pain	Not mentioned	CD3^+^ CD4^+^, CD8^+^ CD2^+^ CD7^+^, CD68^+^, CD20^−^	PTCL-NOS	Proximal ileum	Intestinal perforation	Surgery	100.00%
Bishton MJ (36)	UK	6	40–59	4/2	Dyspepsia, weight loss	Not mentioned	Not mentioned	EATL	Gastrointestinal	Gastrointestinal perforation, bleeding	Chemotherapy + autologous stem cell transplant	66.66%
Novakovic BJ (37)	Slovenia	15	43–73	12/3	Weight loss, abdominal pain, diarrhea	Not mentioned	Not mentioned	EATL, PTCL	Intestinal	Intestinal perforation obstruction	Chemotherapy, surgery, radiotherapy	13.33%
Sato H (38)	Japan	1	92	0/1	Anal bleeding	Not mentioned	CD3^+^, CD5^−^ CD10^−^ CD20^−^, CD56^−^	PTCL-NOS	Rectum	Pleural effusion, ascites	Surgery	0.00%

EATL, enteropathy-associated T-cell lymphoma; ENKL, extranodal NK/T-cell lymphoma nasal type; PTCL-NOS, peripheral T-cell lymphoma, not otherwise specified; ATL, adult T-cell leukemia/lymphoma; ITLPD-GIT, indolent T-cell lymphoproliferative disease of the gastrointestinal tract; ENKTCL, extranodal NK/T-cell lymphoma nasal type; MEITL, monomorphic epitheliotropic intestinal T-cell lymphoma; ALCL, anaplastic large-cell lymphoma; PTCL, peripheral T-cell lymphoma; TIA-1, T-cell intracellular antigen-1; ATLL, adult T-cell leukemia/lymphoma.

Although histopathological examination is essential for definitive diagnosis, obtaining an endoscopic biopsy or even a surgical biopsy during active massive gastrointestinal hemorrhage presents an enormous challenge (27). The visual field is frequently obscured by blood, and the procedure itself carries substantial risks of exacerbating bleeding, infection, hematoma formation, and even perforation (28). Several limitations of endoscopic biopsy exist, such as insufficient sample size, inadequate tissue depth or width, and suboptimal sampling sites. Therefore, when there is a strong suspicion of malignancy despite negative or inconclusive endoscopic biopsies, surgical exploration should be seriously considered. Surgical biopsy yields more substantial tissue samples, improving diagnostic accuracy and enabling thorough evaluation of tumor pathological characteristics, including invasion depth, margin status, and lymphatic vascular invasion. Despite potential risks, surgical intervention remains a justified and valuable diagnostic approach. Fortunately, immunohistochemistry analysis further revealed a markedly elevated Ki-67 proliferation index of 80% and T-lymphocytic origin for neoplastic cells, supporting the diagnosis of aggressive PTCL with intense proliferative activity (29). Notably, pathological results underscore the indispensability of surgical intervention and confirm the appropriateness of clinical stage.

Despite the arduous diagnostic journey, a definitive diagnosis was ultimately established. The strength of the clinical approach relied on several key elements: standardized hemodynamic management, optimized conservative medical therapy, repeated efforts to identify the etiology, and finally, calculated-risk diagnostic surgical exploration. The iterative process of refining the differential diagnosis and adapting management stands out as the cornerstone of the rare case. The absence of any single step would likely have been fatal. Despite the successful discharge after considerable efforts, several important limitations remain. First, the non-specific ulcer presentation, characterized by multiple, irregular, and deep ulcers, always confuses and misleads clinical decision-making. Second, balancing the substantial risks of endoscopic sampling during the active bleeding phase against its potential diagnostic value remains a dilemma. Surgical specimen allows for more comprehensive histopathological assessment, including the lesion’s relationship to surrounding tissues and the extent of invasion, thereby improving diagnostic accuracy. Furthermore, surgery not only serves as a radical treatment but also enables comprehensive genetic sequencing, which is crucial for appropriate targeted therapies. Last but not least, immunohistochemical markers are critical for determining tumor cell origin and type, identifying therapeutic targets, assessing proliferation activity, and evaluating prognosis.

In conclusion, massive gastrointestinal bleeding is a critical condition requiring urgent clinical attention due to the risks of hemorrhagic shock, MODS, and even cardiac arrest. Recurrent bleeding of uncertain etiology should raise suspicion of gastrointestinal tumors, particularly rare lymphoma subtypes such as PTCL. It often masquerades as peptic ulcer disease but is typically refractory to conventional medical therapy, potentially leading to life-threatening complications such as perforation or obstruction. Although CHOP chemotherapy remains the backbone of therapy, the critical condition necessitated diagnostic laparotomy with multidisciplinary collaboration. The efficacy of CHOP in genetically and biologically diverse subtypes remains suboptimal. A genomics‐driven strategy may optimize treatment selection based on tumor biology. Future CHOP‐plus regimens, such as cyclophosphamide, doxorubicin, vincristine, prednisone, etoposide (CHOEP), etoposide, prednisone, vincristine, cyclophosphamide, doxorubicin (EPOCH), and dose‑adjusted EPOCH (DA-EPOCH), may provide personalized treatments (30). Novel agents such as JAK1 inhibitors (31), Histone Deacetylase (HDAC) inhibitors (32), and PI3Kδ inhibitors (33) have exhibited promising efficacy and safety in clinical trials. Achieving more prompt diagnosis of PTCL may facilitate earlier and more accurate treatment initiation, thereby mitigating complication risks and potentially improving overall survival in the future.

## Data Availability

The raw data supporting the conclusions of this article will be made available by the corresponding authors, without undue reservation.

## Supplementary material

Supplementary Figure 1 The PET-CT imaging of the patient diagnosis with PTCL after small intestinal resection. **(A)** The whole body PET-CT image; **(B)** Postoperative linear dense shadow in the ileum and ileocecal region with radioactive concentration SUVmax:3.3; **(C)** Postoperative changes in lower abdomen with radioactive concentration SUVmax:2.8; **(D)** Multiple lymph nodes in the retroperitoneal region without radioactive concentration.
