# Supplementary figures and images for: Rare case report and literature review of peripheral T-cell lymphoma presenting as massive gastrointestinal hemorrhage: an unusual etiology demanding emergency surgical intervention
Source: Front Oncol. 2026 May 8;16:1742112. doi: 10.3389/fonc.2026.1742112 (PMC13193822; doi:10.3389/fonc.2026.1742112)

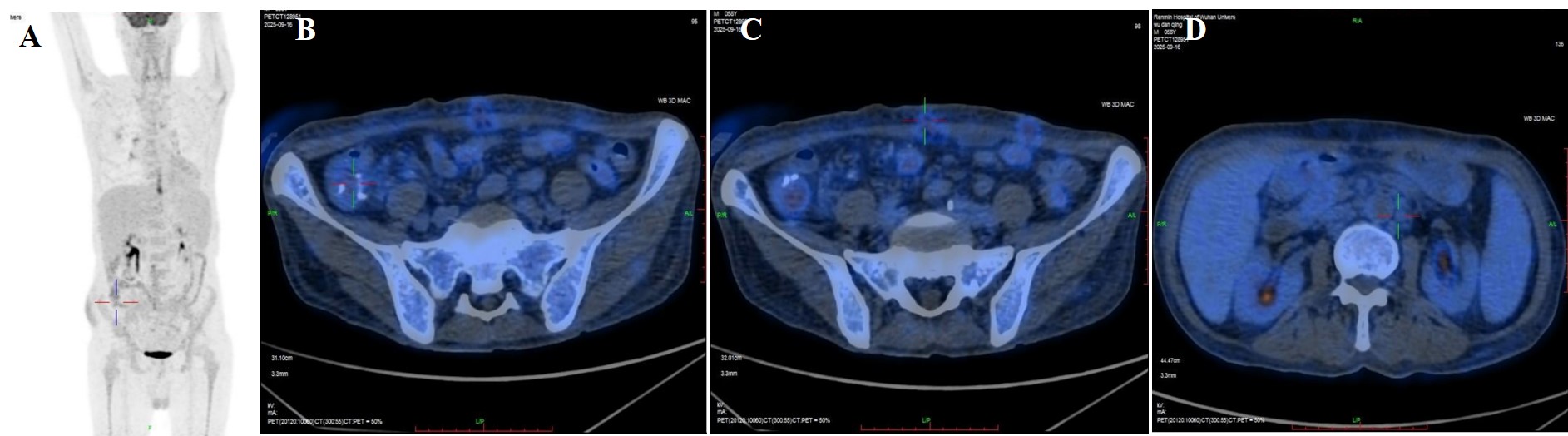

Supplement: Supplementary Figure 1 — The PET-CT imaging of the patient diagnosis with PTCL after small intestinal resection. (A) The whole body PET-CT image; (B) Postoperative linear dense shadow in the ileum and ileocecal region with radioactive concentration SUVmax:3.3; (C) Postoperative changes in lower abdomen with radioactive concentration SUVmax:2.8; (D) Multiple lymph nodes in the retroperitoneal region without radioactive concentration. [file Image1.jpg]
